# Acute carpal tunnel syndrome of the hand following a cat bite

**DOI:** 10.11604/pamj.2015.21.206.7200

**Published:** 2015-07-20

**Authors:** Mohamed Ali Sbai, Slim Dabloun, Sofien Benzarti, Myriam Khechimi, Abdesselem Jenzeri, Riadh Maalla

**Affiliations:** 1 Orthopedic Surgery and Trauma Department, MT Maamouri Hospital, Nabeul, Tunisia; 2 Plastic Surgery Department, La Rabta Hospital, Tunis, Tunisia

**Keywords:** Cat-bite, hand surgery, pasteurella multocida, acute carpal tunnel syndrome, compartment syndrome

## Abstract

Cat bites at the hand are common situation in emergency departments. Neglected or poorly supported, these lesions sometimes lead to serious injuries that may compromise the function of the hand. Pasteurellamultocida is the most offending germ in these lesions, despite their sensitivity to antibiotics; it can sometimes lead to deep infections involving the skin, bones and joints. Acute carpal tunnel syndrome is exceptional after cat bite. We report a case of a 56 Year old female presenting with an acute carpal tunnel syndrome associated with compartment syndrome of the right hand 6 days after a cat bite of her right thumb. The patient was treated by surgery to relieve the median nerve. Microbiology identified PasteurellaMultocida.

## Introduction

Animal bites at hand is a common situation in emergency departments, the neglected cat bite can sometimes cause serious complications like cellulitis, arthritis and osteoarthritis [1]. We report in this work an exceptional case of acute carpal tunnel syndrome with compartment syndrome of the right hand following a neglected cat bite, the objective of this work is to analyze the clinical, microbiological and therapeutic aspects of cat bites. A literature review was made.

## Patient and observation

**Figure 1:**
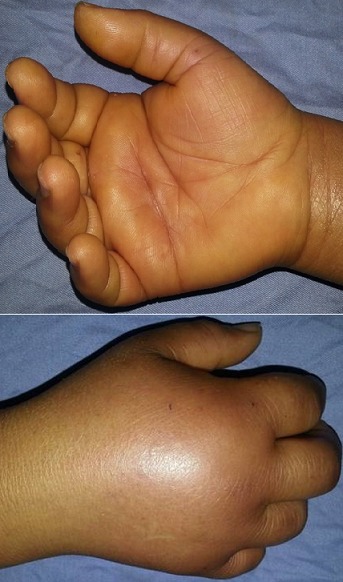
Significant edema of the right hand with a flessum of the fingers

A 56 year old female without any pathological history presented to the emergency department of plastic and hand surgery of La Rabta Hospital in Tunis with a painful swollen right hand evolving for 48 hours. During anamnesis, the patient did not report any recent trauma or inoculation in the hand. But she mentioned that six days earlier, her cat bit her in the thumb. Physical examination showed a significant edema of the right hand with a flessum of the fingers associated to paresthesia in the territory of the median nerve. Passive finger extension was impossible and extremely painful (Figure 1). There was no apparent infection or necrosis of the skin but we noted a high local temperature. Biology showed an elevated rate of white blood cells and a slightly elevated rate of C-reactive protein. The diagnosis of an acute carpal tunnel syndrome associated to a compartment syndrome of the hand was clinically obvious.

**Figure 2:**
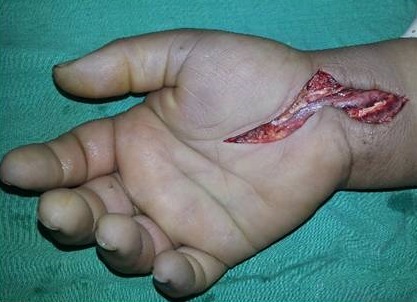
Median nerve presented signs of vascular suffering

**Figure 3:**
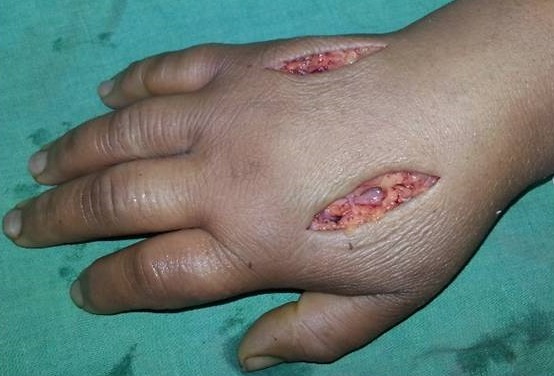
Fasciotomy of the four muscular compartments of the hand

The patient was immediately operated with a large incision of the carpal tunnel that evacuated high pressure viscous yellowish substance from which a sample was taken for microbiology tests. We noticed that the median nerve presented signs of vascular suffering (Figure 2). On the posterior side of the hand, two incisions allowed us to perform fasciotomy of the four muscular compartments of the hand (Figure 3). Immediately, finger extension was easily obtained. The carpal tunnel and the posterior incisions were completely irrigated with saline solution.

**Figure 4:**
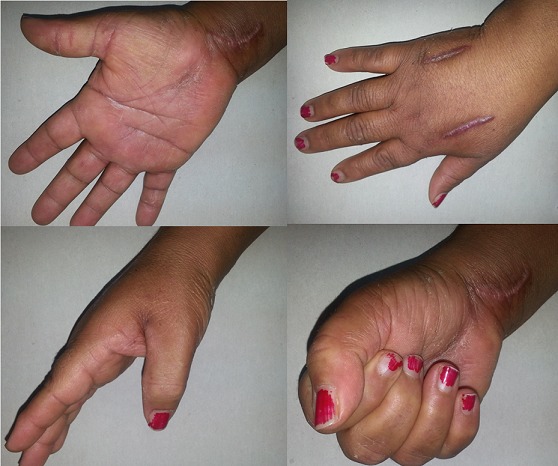
Good finger mobility and a disappearance of paresthesia

After surgery, the patient reported that paresthesia disappeared. A splint was crafted to maintain the hand and wrist in a functional position and limb elevation was advised to limit the edema. The patient was placed under doxycycline and started rehabilitation. After two months of follow up, we noticed a regression of edema, good functional outcome with good finger mobility and a disappearance of paresthesia (Figure 4).

## Discussion

Cat bites account for about 5% of the bite injuries, they represent the second most common mammalian bites [2]. The hand is the most frequently involved site for bite injuries due to the specificity of hand anatomy and the mechanisms of the bites. Even small wounds can lead to aggressive infections, Because of organisms found in cat saliva. Cat bites of the hand particularly represent a potentially devastating problem in terms of wound infection and long-term disability if not treated properly [3]. Puncture wounds caused by cat bites however do result in deeper infections like tenosynovitis [4].

Pasteurella multocida is the major cause of hand infections following cat bites [5–7]. Clinically, pasteurellosis inoculation generally present as an acute loco-regional infection (phlegmon, arthritis etc…) but sometimes the acute episode passes unnoticed and a sub-acute form arises days or weeks after. This particular misleading form is characterized by the absence of suppuration with sometimes a completely healed initial skin lesion at the time of consultation.

Our patient presented an authentic sub-acute form of inoculation pasteurellosis of the hand. The initial lesion, located in the thumb was clearly identified near the metacarpo-phalangeal joint. We looked carefully for signs of arthritis but we haven't found any. As there was no sign of skin necrosis, we thought that it was not pertinent to perform skin debridement of the inoculation site. Surprisingly, it seems that it is not uncommon for the inoculation site to be distant from the symptomatic location [8, 9].

Cat bites of the hand and their associated infections have to be vigilantly monitored and properly managed, by hand surgery experts. Appropriate management of any mammal bite requires evaluation of injured structures, early wound cleansing, and antibio-prophylaxis. Depending on the severity of the injury and it's contamination, wounds may require debridement, empiric antibiotics, and delayed repair or reconstruction [10, 11].

## Conclusion

Proper early treatment of cat bites of the hand usually provides good results. Antibacillary treatment with surgical drainage, debridement and profuse irrigation, proved to be effective. Limb elevation and intensive rehabilitation following a short period of immobilization is necessary.
